# Appendiceal wall thickness and Alvarado score are predictive of acute appendicitis in the patients with equivocal computed tomography findings

**DOI:** 10.1038/s41598-023-27984-8

**Published:** 2023-01-18

**Authors:** Massupa Krisem, Pinporn Jenjitranant, Tharin Thampongsa, Sirote Wongwaisayawan

**Affiliations:** 1 Department of Diagnostic and Therapeutic Radiology, Faculty of Medicine Ramathibodi Hospital, Mahidol University, Bangkok, Thailand; 2 Trauma, Acute Care Surgery and Surgical Critical Care Unit, Department of Surgery, Faculty of Medicine Ramathibodi Hospital, Mahidol University, Bangkok, Thailand

**Keywords:** Diseases, Gastroenterology

## Abstract

Around 8–13% of the patients who underwent CT scan for diagnosis of appendicitis had equivocal CT results. About one-third of these patients had acute appendicitis and this caused diagnostic challenge to the clinicians. This study was conducted to identify clinical and imaging features that were predictive of acute appendicitis in patients who had equivocal CT findings. During January 2015 to June 2021, we retrospectively included 103 consecutive CT scans of adult patients (22 men and 81 women; mean age, 39.1 ± 17.5 years) who had equivocal CT findings of acute appendicitis. Two readers, blinded to the clinical data, independently assessed CT images for the relevant CT findings of appendicitis. Any disagreement between the readers was solved by consensus. The clinical parameters and CT findings were analyzed and compared between the patients who had appendicitis and patients who did not have appendicitis. Thirty-one (30.1%) patients had appendicitis, all of which were non-complicated. The appendiceal wall thickness of ≥ 2 mm and the Alvarado score of ≥ 7 were independent predictors of appendicitis with adjusted odds ratios (ORs) of 2.76 (95% CI, 1.09–7.02) and 1.47 (95% CI, 1.12–1.94), respectively. The maximal appendiceal diameter was higher in the appendicitis group (7.2 ± 1.2 mm vs. 6.5 ± 1.0 mm), but not predictive of appendicitis. The rest of the clinical parameters and CT findings, including mucosal hyperenhancement, periappendiceal fat reticulation, thickening of peritoneal reflection, appendicolith, focal cecal thickening, and content in appendiceal lumen showed no significant difference between two groups. The appendiceal wall thickness and the Alvarado score were able to predict appendicitis in patients who had equivocal CT findings.

## Introduction

Acute appendicitis is the most common acute abdominal pain in the emergency department requiring emergency abdominal surgery. Computed tomography (CT) is considered imaging of choice for evaluation of the patients due to the high sensitivity and specificity over 95%^1–4^. CT can also assess the complications, and provides alternative diagnoses of non-surgical conditions such as pelvic inflammatory disease, omental infarction, epiploic appendagitis, enterocolitis and diverticulitis^5–7^. The CT criteria for appendicitis typically comprise of enlarged appendiceal diameter of more than 6 mm, wall thickening and hyperenhancement, and periappendiceal fat stranding^8–11^. However, CT diagnosis of appendicitis can be very challenging in thin patients and during early course of appendicitis, which only minimal change of appendix and periappendiceal tissues are present. It is estimated that around 8–13% of the patients with right lower quadrant (RLQ) pain have equivocal CT findings and up to 30% of these patients being later diagnosed with appendicitis^12–14^.

There are only a few data on the predictors of acute appendicitis in the patients who have equivocal CT findings^12,15^. Therefore, this study was conducted to determine the predictive factors of acute appendicitis in patients who have equivocal CT findings.

## Methods

### Study population

Using radiology database system, we retrieved a cohort of patients who received CT scan for clinical suspicion of appendicitis during January 2015 to June 2021. Our CT reporting system expressed the likelihood of appendicitis in terms of CT certainty score, using 5-point ranking scale ranging from 1 to 5 (certainty score 1, definitely absent; certainty score 2, unlikely; certainty score 3, equivocal; certainty score 4, likely; and certainty score 5, definitely present)^16^. Certainty score 1 and 2 were considered negative for appendicitis while certainty score 4 and 5 were considered positive for appendicitis. Certainty score 3 was deemed indeterminate or equivocal for appendicitis. A total of 105 adult patients (greater than or equal to 15 years of age) who had equivocal CT findings for appendicitis were enrolled. One of the authors retrospectively reviewed the electronic medical records for the demographic data, presenting signs and symptoms, relevant laboratory results, Alvarado score^17^, and pain score. Surgical pathology results were used as a reference standard in the patients who had surgery. In the patients without surgery, the clinical follow-up records were reviewed up to three months after the initial visit. Two patients were excluded from the study because of the inadequate CT image quality. The final cohort consisted of 103 adult patients with 103 CT scans.

### CT technique

At the authors’ institution, the graded-compression ultrasound (US) was used as a first-line imaging modality in all patients with suspected acute appendicitis. Second-line CT scan was performed in the patients who had indeterminate or nondiagnostic US results, or the appendices were not visualized on the US.

CT scan was performed in a supine position. The CT scanning parameters were as follows: 120 kVp, automated tube current modulation, 3-mm section collimation, and a section interval of 3 mm. A solution of 25 ml of iodinated contrast media mixed with 1000 ml of normal saline was given rectally. After that, intravenous (IV) non-ionic iodinated contrast media was given via a power injector at a rate of 2–3.3 ml/sec followed by a saline chaser. The total amount of IV contrast media was calculated by the body weight of the patients (ranging from 450 to 555 mgI/kg). A single-phase scan was initiated at 120 s after the initiation of IV contrast administration. The scan coverage was from the superior endplate of L3 to the pubic symphysis. The axial CT images were reconstructed into sagittal and coronal images with a slice thickness of 3 mm.

### Image processing and analysis

All CT images were transferred via the Picture Archiving and Communications System (PACS), using a DICOM Conformance (Synapse version 3.2.0, FUJIFILM Medical Systems USA’s Synapse® PACS System, USA).

Two emergency radiologists separately assessed CT scans in random order. Both of whom were blinded to the patient data and the final diagnosis, except for the clinical indication of suspected appendicitis. Both readers were asked to review the CT images for the relevant findings of appendicitis, including the maximal diameter of the appendix, single wall thickness, mucosal hyperenhancement, periappendiceal fat reticulation, enhancing and thickening of peritoneal reflection, appendicolith, focal cecal thickening at the base, and content in the appendiceal lumen. Any discrepancies of CT findings between the two readers were solved by consensus.

### Statistical analysis

The statistical analyses were performed by using STATA version 17.0 (Stata Corp, College Drive, Texas, USA) statistical software. The demographic data, presenting signs and symptoms, and CT parameters were expressed as percentage, mean ± standard deviation, and median and range. Univariate and stepwise multivariate logistic regression analysis was used to determine the independent factors for predicting appendicitis. The cut-off values were calculated based on the highest sensitivity and specificity. The intraclass correlation coefficient (ICC) and Cohen’s kappa coefficient were used to determine interobserver agreement. A p-value of less than 0.05 was considered to indicate a significant difference.

### Ethics approval and consent to participate

This retrospective cross-sectional study was conducted at a university hospital. The study was approved by the Human Research Ethics Committee, Faculty of Medicine Ramathibodi Hospital, Mahidol University (reference number: MURA2020/2001). All methods were performed in accordance with the Declaration of Helsinki. The informed consent was waived by the Human Research Ethics Committee, Faculty of Medicine Ramathibodi Hospital, Mahidol University.

## Results

Eighty-one (78.6%) women and 22 (21.4%) men were included in the final cohort. The mean age was 39.1 ± 17.5 years (range, 15–80 years). Thirty-one (30.1%) patients had acute appendicitis, all of which were non-complicated type. The alternative diagnoses of the rest patients were as follows: enterocolitis (n = 17), pelvic inflammatory disease (n = 6), complicated ovarian cyst or tumor (n = 7), endometriosis (n = 3), acute genitourinary tract infection (n = 4), non-specific abdominal pain (n = 33), peptic ulcer perforation (n = 1) and infected lymphocele (n = 1).

Table 1 compares the clinical parameters between appendicitis and non-appendicitis groups. The Alvarado score was significantly higher in the patients with appendicitis (6.1 vs. 4.8; p = 0.002). The other demographic parameters and clinical signs and symptoms did not significantly differ between the two groups.

**Table 1:** Comparison of clinical parameters between appendicitis and non-appendicitis groups.

Clinical parameters	Appendicitis (N = 31)	Non-appendicitis (N = 72)	p-value
**Gender**			0.47
Male	8 (25.8%)	14 (19.4%)	
Female	23 (74.2%)	58 (80.6%)	
Age, mean ± SD	37 ± 14.3	40 ± 18.8	0.44
Duration of illness (hours), median (range)	10 (2, 120)	17.5 (1, 168)	0.09
**Clinical signs and symptoms**			
Fever	10 (32.3)	27 (37.5)	0.78
RLQ pain	29 (93.5)	71 (98.6)	1.00
Migratory pain	14 (45.2)	21 (29.2)	0.07
Anorexia	14 (45.2)	29 (40.3)	0.46
Nausea/vomiting	14 (45.2)	33 (45.8)	0.82
WBC count, median (range)	12,800 (5100, 21,500)	11,100 (3400, 105,000)	0.22
Alvarado score, mean ± SD	6.1 ± 1.6	4.8 ± 2.0	0.002
Pain score, mean ± SD	6.3 ± 2.0	6.2 ± 2.5	0.89

*RLQ:* right lower quadrant, *WBC:* white blood cell.

**Figure 1:**
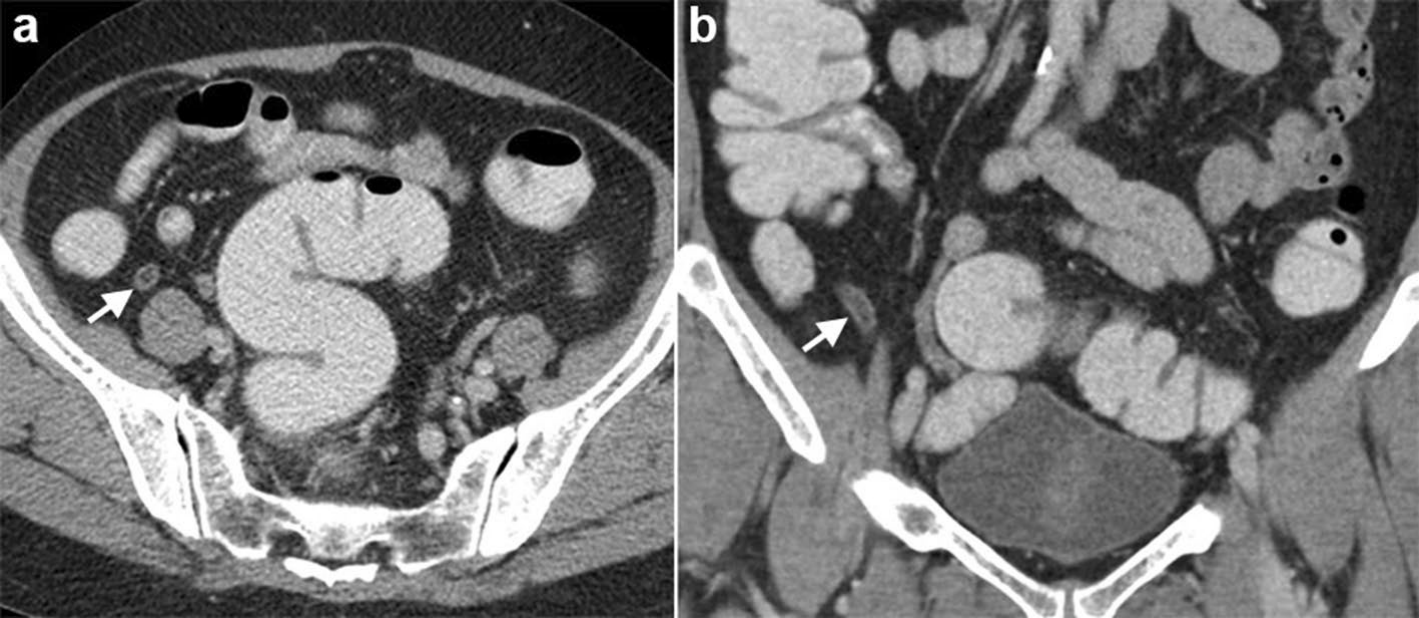
Acute appendicitis in a 65-year-old female with Alvarado score of 7. Cross-sectional (**a**) and coronal (**b**) computed tomography scan showing enlarged appendiceal diameter and wall thickening (arrows).

The appendicitis group had a larger maximal diameter (p = 0.005) and thicker appendiceal wall (p = 0.014) than the patients without appendicitis (Fig. 1). There was moderate agreement for measurement of maximal diameter and good agreement for measurement of wall thickness. Variable agreement of CT findings between two readers was noted, with peritoneal thickening had the lowest agreement and gas in appendix lumen had the highest agreement. The data is shown in Table 2.

**Table 2:** Comparison of CT findings between appendicitis and non-appendicitis groups.

CT findings	Appendicitis (N = 31)	Non-appendicitis (N = 72)	p-value	ICC	Cohen’s kappa
Maximal appendiceal diameter, mean ± SD	7.2 ± 1.2	6.5 ± 1.0	0.005	0.698 (0.553–0.795)	–
Appendiceal wall thickness, mean ± SD	1.9 ± 0.5	1.6 ± 0.5	0.014	0.872 (0.811–0.914)	–
Mucosal hyperenhancement	12 (38.7%)	27 (37.5%)	0.908	–	0.37 (0.18–0.56)
Fat reticulation	6 (19.4%)	21 (29.2%)	0.299	–	0.24 (0.08–0.41)
Thickening of peritoneal reflection	5 (16.1%)	12 (16.7%)	0.946	–	0.16 (0.03–0.29)
Appendicolith	2 (6.5%)	3 (4.2%)	0.636	–	0.65 (0.46–0.84)
Focal cecal thickening	11 (35.5%)	17 (23.6%)	0.214	–	0.25 (0.08–0.41)
Gas in lumen of appendix	20 (64.5%)	42 (58.3%)	0.557	–	0.94 (0.75–1.00)
Contrast in lumen of appendix	16 (51.6%)	38 (52.8%)	0.914	–	0.55 (0.34–0.76)

*ICC:* intraclass correlation coefficient.

The single wall thickness of the appendix of ≥ 2 mm and the Alvarado score of ≥ 7 were only two independent predictors of appendicitis on multivariate regression analysis, with adjusted odds ratios (ORs) of 2.76 (95% CI, 1.09–7.02) and 1.47 (95% CI, 1.12–1.94), respectively (Table 3).

**Table 3:** Univariate and multivariate analyses.

Parameters	Univariate analysis		Multivariate analysis	
	OR	p-value	OR	p-value
Alvarado score	1.48 (1.13–1.93)	0.004	1.47 (1.12–1.94)	0.006
Appendiceal wall thickness	2.81 (1.21–6.54)	0.040	2.76 (1.09–7.02)	0.033
Maximal appendiceal diameter	1.83 (1.18–2.83)	0.007	–	–

*OR:* odds ratio.

## Discussion

Dealing with patients who have equivocal CT scan for appendicitis is very challenging because the chance of appendicitis in these patients could be as high as 30%^12^. Targeted US and reassessment of CT images can improve accuracy of diagnosis of appendicitis in these patients^18,19^. In our institution, targeted US after equivocal CT scan is not routinely performed, especially during off-hour period. Therefore, simple clinical scoring system, such as Alvarado score, and CT reassessment may provide useful information to clinicians. We found that appendiceal wall thickness of ≥ 2 mm and the Alvarado score of ≥ 7 were predictive of acute appendicitis in patients who had equivocal CT findings.

The appendiceal wall thickening reflects mural inflammation of the appendix and represents early change in course of disease which may help pick up cases with early appendicitis. A previous study had showed modest sensitivity (51.9–66.7%) and high specificity (85.2–87.5%) of CT reassessment in diagnosis of appendicitis in patients with equivocal CT findings^18^. However, in their study, the appendiceal wall thickening was subjectively determined by comparing to the normal bowels. This subjective measurement is easy and simple to perform, but there could be potential error in cases with collapsed bowel loops. Kim et al. showed that the appendiceal wall enhancement, intraluminal air in appendix lumen, coexistent inflammatory lesion and appendiceal wall thickening can differentiate appendicitis in patients with equivocal findings^19^. Park et al. reported that 78% of the patients with appendicitis had appendiceal wall thickness of ≥ 2 mm as compared to 67% of the patients without appendicitis, although this observation did not reach statistical significance^20^. Our result confirmed their observation with good level of agreement. In our study, patients who had appendiceal wall thickness ≥ 2 mm were 2.76 times more likely to have appendicitis. Nevertheless, it was still difficult to determine actual wall thickness in cases with collapsed appendiceal lumen. US can play complimentary role to solve this issue. The presence of non-compressibility and increased Doppler flow on appendix wall are useful signs to suggest appendicitis, but this was not investigated in our study^21^. Another limitation is that, to accurately assess appendiceal wall thickness, the administration of IV contrast medium helps improve the visualization and evaluation. Therefore, this CT finding may be of limited value in non-contrast scan.

The Alvarado score is a simple, well-known and easy-to-use clinical scoring system^17^. Our result found that in patients with Alvarado score ≥ 7 (high-risk), they were 1.47 times more likely to have appendicitis even their CT finding did not show typical signs of appendicitis. Chae et al. reported moderate accuracy of Alvarado score (area under the curve (AUC) = 0.698 (95% CI, 0.627–0.762)) in diagnosis of appendicitis in 189 patients with equivocal CT findings^22^. Kang et al. also reported that Alvarado score had an AUC of 0.695 (95% CI, 0.633–0.752) in predicting appendicitis in patients with equivocal CT findings^23^. This moderate accuracy was probably because of the early stage of appendicitis. The majority of patients had early stage of appendicitis and their symptoms were not characteristic of appendicitis, thus having intermediate Alvarado score. This result emphasizes the importance of clinical and radiological correlation for the decision-making, as the positive predictive value of the CT scan is directly proportional to the clinical pretest probability. A recent meta-analysis reported significant odds ratio of all parameters of Alvarado score for predicting appendicitis^24^ but this was not investigated in our study.

Our result indicated that the maximal appendiceal diameter was larger in the appendicitis group, but not reaching statistical significance on multivariate analysis. Appendiceal enlargement can be an isolated finding of appendicitis and was reported in up to 48% of symptomatic individuals^12^. The diameter cut-off of 6 mm was helpful to diagnose appendicitis^12,20,25^. In their study, appendicitis was not found in any patients who had appendiceal diameter less than 6 mm. On the contrary, appendicitis was present in 35.5% of patients who had isolated appendiceal enlargement ≥ 6 mm^12^. However, this 6-mm threshold was originally derived from US publications which used graded-compression technique during scan^26,27^. Therefore, it is difficult to extrapolate to CT scan, in which appendix is not compressed during the acquisition. Several prior studies have shown that normal appendiceal diameter can be greater than 6 mm in up to 61% of individuals^28–31^. The content in the appendiceal lumen, such as fluid, gas or feces may increase appendiceal diameter^28^. Therefore, the measurement of appendiceal diameter is not always a good indication of appendicitis. Another explanation is that our study included only the patients with equivocal CT findings, most of whom had borderline appendiceal diameter. The patients who had normal or frankly large appendiceal diameter would be unequivocally diagnosed with either normal appendix or appendicitis, and thus, excluded from our cohort.

Several limitations should be mentioned. The data acquisition was limited owing to the retrospective nature of the study. The population size was relatively small. Our results should be further validated with a larger prospective study. Twenty-seven of 72 (37.5%) patients in non-appendicitis group received either oral or IV antibiotics. The success rate of antibiotic therapy alone in uncomplicated appendicitis is around 60–72.7%^4,32,33^. Therefore, the true incidence of appendicitis in patients with equivocal CT findings might have been underestimated. There was a variable time interval from the CT scan to the operation which could affect the CT performance. The patients had second-line CT scans following the inconclusive US results. This approach scheme may be subject to selection bias, as the patients with a high likelihood of appendicitis from the US would directly go to the operating room without the need for CT. The proportion of female patients in our cohort were higher than male. This observation may be consequence of using conditional CT scheme. The attending physicians might have lower threshold to perform CT scan in female patients who had inconclusive US, in order to rule out both appendicitis and acute gynecologic condition. Moreover, our institutional protocol mandates the use of rectal contrast administration, which might have influenced the interpretation of CT findings. These could limit the generalization of our results.

## Conclusions

The appendiceal wall thickness and the Alvarado score were able to predict acute appendicitis in the patients who had equivocal CT findings. These two factors are not too difficult to assess in routine practice. Therefore, the radiologists, surgeons and emergency physicians could improve the diagnostic accuracy of appendicitis by using these criteria.

## Data Availability

The dataset used and/or analyzed during the current study are available from the corresponding author on reasonable request.

## Abbreviations

CT: Computed tomography
RLQ: Right lower quadrant
US: Ultrasound
IV: Intravenous
PACS: Picture archiving and communications system

## References

[1] Rud B, Vejborg TS, Rappeport ED, Reitsma JB, Wille-Jorgensen P (2019). Computed tomography for diagnosis of acute appendicitis in adults. Cochrane Database Syst. Rev..

[2] Wongwaisayawan S, Tangkittithaworn P, Klawandee S, Prapruttam D (2021). Diagnostic performance and reliability of the standardized computed tomography reporting system for acute appendicitis: Experience in a tertiary care adacemic center. J. Med. Assoc. Thai..

[3] Arruzza E, Milanese S, Li LSK, Dizon J (2022). Diagnostic accuracy of computed tomography and ultrasound for the diagnosis of acute appendicitis: A systematic review and meta-analysis. Radiography (Lond)..

[4] Moris D, Paulson EK, Pappas TN (2021). Diagnosis and management of acute appendicitis in adults: A review. JAMA.

[5] Kambadakone AR, Santillan CS, Kim DH, Fowler KJ, Birkholz JH, Expert Panel on Gastrointestinal Imaging (2022). ACR appropriateness criteria® right lower quadrant pain: 2022 update. J. Am. Coll. Radiol..

[6] Morley EJ, Bracey A, Reiter M, Thode HC, Singer AJ (2020). Association of pain location with computed tomography abnormalities in emergeny department patients with abdominal pain. J. Emerg. Med..

[7] Pooler BD, Lawrence EM, Pickhardt PJ (2012). Alternative diagnoses to suspected appendicitis at CT. Radiology.

[8] Hunsaker, J. C., Aquino, R., Wright, B., Kobes, P., Kennedy, A., & Dunn, D. Review of appendicitis: Routine, complicated, and mimics. Emerg. Radiol. 2022. Online ahead of print. 10.1007/s10140-022-02098-2 36376643

[9] Kim HY, Park JH, Lee YJ, Lee SS, Jeon JJ, Lee KH (2018). Systematic review and meta-analysis of CT features for differentiating complicated and uncomplicated appendicitis. Radiology.

[10] Moteki T, Horikoshi H (2007). New CT criterion for acute appendicitis: Maximum depth of intraluminal appendiceal fluid. Am. J. Roentgenol..

[11] Quadri R, Vasan V, Hester C, Porembka M, Fielding J (2019). Comprehensive review of typical and atypical pathology of the appendix on CT: Cases with clinical implications. Clin. Imaging..

[12] Daly CP, Cohan RH, Francis IR, Caoili EM, Ellis JH, Nan B (2005). Incidence of acute appendicitis in patients with equivocal CT findings. Am. J. Roentgenol..

[13] Simianu VV, Shamitoff A, Hippe DS, Godwin BD, Shriki JE, Drake FT (2017). The reliability of a standardized reporting system for the diagnosis of appendicitis. Curr. Probl. Diagn. Radiol..

[14] Weyant MJ, Eachempati SR, Maluccio MA, Rivadeneira DE, Grobmyer SR, Hydo LJ (2000). Interpretation of computed tomography does not correlate with laboratory or pathologic findings in surgically confirmed acute appendicitis. Surgery..

[15] Hong HS, Cho HS, Woo JY, Lee Y, Yang I, Hwang JY (2016). Intra-appendiceal air at CT: Is it a useful or a confusing sign for the diagnosis of acute appendicitis?. Korean J. Radiol..

[16] Godwin BD, Drake FT, Simianu VV, Shriki JE, Hippe DS, Dighe M (2015). A novel reporting system to improve accuracy in appendicitis imaging. Am. J. Roentgenol..

[17] Alvarado A (1986). A practical score for the early diagnosis of acute appendicitis. Ann. Emerg. Med..

[18] Sim JY, Kim HJ, Jang SK, Yeon JW, Jeon BG, Ha YR (2019). Value of additional ultrasound examination in patients with equivocal computed tomography findings of acute appendicitis: Comparison with computed tomography reassessment. J. Med. Ultrasound..

[19] Kim HC, Yang DM, Kim SW, Park SJ (2012). Reassessment of CT images to improve diagnostic accuracy in patients with suspected acute appendicitis and an equivocal preoperative CT interpretation. Eur. Radiol..

[20] Park G, Lee SC, Choi BJ, Kim SJ (2014). Stratified computed tomography findings improve diagnostic accuracy for appendicitis. World J. Gastroenterol..

[21] Kim MS, Kwon HJ, Kang KA, Do IG, Park HJ, Kim EY (2018). Diagnostic performance and useful findings of ultrasound re-evaluation for patients with equivocal CT features of acute appendicitis. Br. J. Radiol..

[22] Chae MS, Hong CK, Ha YR, Chae MK, Kim YS, Shin TY (2017). Can clinical scoring systems improve the diagnostic accuracy in patients with suspected adult appendicitis and equivocal preoperative computed tomography findings?. Clin. Exp. Emerg. Med..

[23] Kang HJ, Kang H, Kim B, Chae MS, Ha YR, Oh SB (2019). Evaluation of the diagnostic performance of a decision tree model in suspected acute appendicitis with equivocal preoperative computed tomography findings compared with Alvarado, Eskelinen, and adult appendicitis scores: A STARD compliant article. Medicine (Baltimore).

[24] Kinesya E, Cintya EP, Dorothy MJ, Ennaidi NN, Rusti HF, Mannagalli Y (2022). Diagnostic accuracy of Alvarado score components in patients with appendicitis: Systematic review and meta-analysis approach. Health Sci. Rev..

[25] Tames AC, Yamauchi FI, Castro A, Amoedo CDM, Cardoso EF, Baroni RH (2019). Morphologic criteria of vermiform appendix on computed tomography and a possible risk of developing acute appendicitis. Radiol. Bras..

[26] Rioux M (1992). Sonographic detection of the normal and abnormal appendix. Am. J. Roentgenol..

[27] Jeffrey RB, Laing FC, Townsend RR (1988). Acute appendicitis: Sonographic criteria based on 250 cases. Radiology.

[28] Tamburrini S, Brunetti A, Brown M, Sirlin CB, Casola G (2005). CT appearance of the normal appendix in adults. Eur. Radiol..

[29] Charoensak A, Pongpornsup S, Suthikeeree W (2010). Wall thickness and outer diameter of the normal appendix in adults using 64 slices multidetector CT. J. Med. Assoc. Thai..

[30] Moskowitz E, Khan AD, Cribari C, Schroeppel TJ (2019). Size matters: Computed tomographic measurements of the appendix in emergency department scans. Am. J. Surg..

[31] Webb EM, Wang ZJ, Coakley FV, Poder L, Westphalen AC, Yeh BM (2010). The equivocal appendix at CT: Prevalence in a control population. Emerg. Radiol..

[32] Podda M, Gerardi C, Cillara N, Fearnhead N, Gomes CA, Birindelli A (2019). Antibiotic treatment and appendectomy for uncomplicated acute appendicitis in adults and children: A systematic review and meta-analysis. Ann. Surg..

[33] Salminen P, Paajanen H, Rautio T, Nordstrom P, Aarnio M, Rantanen T (2015). Antibiotic therapy vs appendectomy for treatment of uncomplicated acute appendicitis: The APPAC randomized clinical trial. JAMA.

